# Improving young children’s handwashing behaviour and understanding of germs: The impact of A Germ’s Journey educational resources in schools and public spaces

**DOI:** 10.1371/journal.pone.0242134

**Published:** 2020-11-23

**Authors:** Sarah Younie, Chloe Mitchell, Marie-Josee Bisson, Sapphire Crosby, Anuenue Kukona, Katie Laird

**Affiliations:** 1 Institute for Research in Criminology, Community, Education and Social Justice, De Montfort University, Leicester, United Kingdom; 2 Institute for Psychological Science, De Montfort University, Leicester, United Kingdom; 3 Infectious Disease Research Group, Leicester Institute for Pharmaceutical Innovation, De Montfort University, Leicester, United Kingdom; National Institute for Infectious Diseases Lazzaro Spallanzani-IRCCS, ITALY

## Abstract

**Context:**

Effective handwashing can prevent the spread of germs, including Covid-19. However, young children can lack a fundamental understanding of germ transfer. A Germ’s Journey educational resources were designed to support young children in learning about germs and handwashing. These resources include a book, website, song, online games and glo-gel activities that are informed by a behaviour change model.

**Research gap:**

Prior research has not evaluated the impacts of these resources on behavioural outcomes.

**Purpose of the study:**

Two intervention studies evaluated the impacts of these resources on both knowledge and behavioural outcomes.

**Method:**

In Study 1, children (n = 225) were recruited from four schools and randomly assigned by classrooms to participate in a multicomponent intervention (vs. control). In Study 2, children (n = 104) were recruited from a museum and randomly assigned to participate in a song intervention (vs. control). Trained observers recorded participants’ engagement in six handwashing behaviours and their understanding of germs. These behavioural and knowledge outcomes were analysed using regression and related analyses.

**Results:**

In Study 1, significant improvements were observed between baseline and follow up in the intervention group for both behavioural scores (*Est* = 0.48, *SE* = 0.14, *t* = 3.30, *p* = 0.001) and knowledge scores (*Est* = 2.14, *SE* = 0.52, *z* = 4.11, *p* < 0.001), whereas these improvements were not observed in the control group (*t*s < 1). In Study 2, the intervention group had significantly higher behavioural scores compared to the control group (*Est*. = -0.71, *SE* = 0.34, *t* = -2.07, *p* = 0.04).

**Conclusion:**

This research demonstrates that specifically designed hand hygiene educational resources can improve handwashing practice and understanding in young children, and could lead to the reduction of the transmission of disease within this group.

## Introduction

Handwashing is an increasingly significant issue. The relationship between handwashing and the prevention of childhood illnesses and the spread of the 2019 novel coronavirus (Covid-19) is particularly important [1, 2]. Both the World Health Organisation (WHO) and Centers for Disease Control and Prevention (CDC) advocate the use of non-pharmaceutical interventions such as handwashing to prevent the spread of infectious disease [3, 4]. According to Randle and colleagues [5], approximately one-third of infections are preventable by practising correct handwashing. Research has shown that school-based interventions are one of the most effective methods of encouraging correct handwashing behaviour in children [6]. Randle and colleagues [5] state that educational interventions have the potential to increase hand hygiene (HH) and reduce the transmission of infections. A systematic review of the published literature suggested that efficacious handwashing interventions aimed at children have the potential to lead to significant health improvements [7]. However, prior research found that there were few handwashing educational resources aimed at children in the Early Years Foundation Stage (EYFS) [8], which includes children up to five years of age within the United Kingdom. The purpose of the current study was to evaluate A Germ’s Journey educational resources, which were designed to support young children in learning about germs and handwashing. The current study measured the impact of these resources on both knowledge and behavioural outcomes; the latter has not been addressed in previous research and reflects an important research gap. As the global coronavirus pandemic attests, the need to address infection prevention is paramount and reducing contagion through effective handwashing is part of the solution.

The current Covid-19 pandemic means that behaviour change in relation to handwashing is of the utmost importance. Young children are a vulnerable population group in relation to both the spreading and contracting of infectious disease. Communicable diseases present as a substantial cause of morbidity among this population group in the United Kingdom as a developed country [9–11]. In addition to their immature immune systems [12], behavioural factors such as a tendency to explore objects with their hands and mouths make children particularly susceptible to and a good transmitter of communicable illnesses [13]. This makes schools a hotspot for the transmission of pathogens and can result in several deleterious outcomes such as high rates of illness related absenteeism, secondary transmission of infections to family members, increased health care costs and an increase in the use of antimicrobial medication and subsequent antibiotic resistance [14–17]. The concept of children as ‘super spreaders’ became prominent during the coronavirus pandemic, leading to mass school closures across an estimated 186 countries [18], and highlighting the role of children in the transference of viruses through close contact and lack of effective HH.

Early childhood offers an advantageous time to target HH behaviours before habits are firmly grounded [19]. The school environment can also aid in the facilitation of behavioural change by supporting children in learning about the concept of the invisible germ and about *why* handwashing is essential. For several decades, the WHO have emphasised the influential role of schools in promoting health [20]. In the United Kingdom, the EYFS statuary framework, which covers children up to five years of age, states that children should be capable of managing their own basic hygiene effectively [21]. Implementation of an efficacious HH intervention in EYFS and related settings offers the potential to equip young children with an essential skill for life, which they can also pass on to family members [22, 23].

Whilst multiple HH interventions for children exist, research investigating their utility and efficacy is limited in a number of respects [24–26]. One concern is that many of the existing interventions are atheoretical [24]. Utilising behavioural theories and frameworks in the design, implementation and evaluation of behaviour change interventions offers a number of potential advantages [27]. Theoretically based interventions that incorporate a range of behaviour change techniques have consistently been shown to be more effective at changing health behaviours, and can aid in understanding how components of interventions contribute to their overall success [28–31].

Perhaps owing to their atheoretical nature, some HH interventions have oversimplified behaviours, focussing on one approach such as persuasive fear appeals or information provision alone [24]. HH behaviours are complex and multifactorial. Behavioural determinants consist of both deliberative and automatic influences [32], such as psychological, social, emotional, environmental, and habitual factors [33, 34]. An individual’s propensity to engage in effective HH behaviours is likely to depend on a variety of these factors, and different individuals in a population group are also likely to depend on differing factors [35]. Due to the complex nature of HH and behaviour change in general, HH interventions that aim to target multiple behavioural determinants are likely to maximise benefits.

The Capability, Opportunity, Motivation, Behaviour (COM-B) model, which is a part of a wider framework known as the Behaviour Change Wheel (BCW), is consistent with this perspective. The BCW [36] is derived from 19 behaviour change frameworks, providing a comprehensive and systematic framework for intervention development that is linked to behaviour change theories and applies to a broad range of behaviours [37, 38]. This framework has been applied in multiple areas of public health concern, such as smoking cessation [39] and physical activity [40]. The COM-B model is at the core of the framework, positing that behaviour (B) occurs as an interaction between an individual’s psychological and physical capability (C), physical and social opportunities (O) and automatic and reflective motivations (M) to engage in the desired behaviour [34]. The COM-B model also identifies behavioural targets. Interventions that target multiple components of the COM-B are predicted to have more favourable outcomes than interventions that target only one element [41]. The second layer of the BCW consists of nine intervention functions, which focus on how the intervention can change behaviour [42]. Finally, the outer layer identifies policy types for delivering the intervention. Thus, the BCW provides a theoretically motivated and empirically grounded framework for designing a multicomponent HH intervention that targets several determinants of HH behaviour.

Finally, a further concern with regards to prior HH interventions relates to the methodological design of their evaluations [26]. The outcome measures of prior studies vary from (e.g., retrospective) self-report measures, to measures such as illness related absences from schools, where direct causality of the intervention can be difficult to infer [e.g., 43, 44], and it can be unclear whether the intervention influenced HH [26]. This has led to calls for studies to prioritise observable HH behavioural measures [35]. Furthermore, those studies that do use observations tend to target and observe the frequency of handwashing before or after key events such as food consumption or using the toilet, without explaining or providing training on HH [24]. Given published research suggesting that HH quality is important in combatting the spread of germs including coronavirus [45], and several theories highlighting the importance of coping appraisals, such as self-efficacy and response efficacy in instigating behavioural change (e.g., Protection motivation theory [46]), interventions would benefit from targeting and assessing their success based on HH quality.

### The intervention

The current research focused on children aged 4–5 years (i.e., EYFS key stage in the UK), who reflect a vulnerable yet challenging population group in the context of HH. Utilising the COM-B model (see Table 1), potential barriers to effective HH practices in young children include a lack of knowledge and understanding about germ transfer and effective handwashing techniques (psychological capability), access to handwashing facilities (physical opportunity), poor social modelling of handwashing (social opportunity) and a lack of awareness regarding their susceptibility and the consequences of germs and inadequate handwashing (motivation). Simultaneously, building on limitations of prior interventions, key behavioural targets include young children’s handwashing quality (e.g., covering key areas such as soap use) and understanding of germs. The current research focused on A Germ’s Journey educational resources [6, 47], which were developed by an interdisciplinary research team and were co-created with scientists, educators and children, comprising of a book [48], website, song, online games and glo-gel activities. These resources specifically target the key areas of the COM-B model. The Germ’s Journey resources that are used in the intervention are available at-the-point-of-access on the website (http://germsjourney.com/learningresources.html) under a Creative Commons License (CC-BY), including a structured, detailed lesson plan of the workshops so that they can easily be replicated.

**Table 1:** COM-B analysis of the intervention.

Component	Sub-component	Relevance to handwashing in children	Integration into intervention
Capability	Psychological	Knowledge of handwashing techniques.	Book
			Song
		Confidence in handwashing technique.	Online Games
			Glo-gel Activity
	Physical	Physical ability and skills to wash hands	Glo-gel Activity
Opportunity	Physical	Access to handwashing facilities	N/A
		Location of soap and taps	
		Access to sinks during the school day.	
	Social	Role models	Song/ video
			Glo-gel activity
Motivation	Reflective	Perceived benefits of handwashing	Book
		Perceived costs of poor hand hygiene	
		Perceived susceptibility of adverse outcomes.	
		Perceived severity of adverse outcomes.	
	Automatic	Disgust	Book
		Fear	Online games

#### Book: Illustrated children’s book using thermochromic ink

The first resource is the book, *A Germ’s Journey* [48]. The book aims to educate children about the presence of germs, the transmission of germs, the consequences of poor HH and how to evade the threat of germs via handwashing. The book targets key areas of the COM-B model through the intervention functions of education and persuasion. Psychological capability is targeted by teaching children about the transmission of germs and how to get rid of them, thus providing them with knowledge of how to avert the threat of illness. Additionally, a unique aspect of the book is the use of thermochromic ink, which ‘hides’ the germs on the children’s hands in the pictures, but when rubbed away, reveals hidden germs underneath the black ink; the use of friction provides heat to ‘dissolve’ the ink. This feature was employed to increase children’s perceptions of susceptibility and exposure to ‘invisible germs’, targeting the reflective motivation element of the COM-B model. Furthermore, to target automatic motivation, the book also features an image of an illustrated character vomiting and an image of the same character touching the toilet to evoke the emotion of disgust, which has been highlighted as being one of the strongest determinants of behaviour change in prior HH interventions [49, 50].

#### Song: A step-by-step action song

The second resource is a song and associated video that the research team created collaboratively with the Thinktank Birmingham Science Museum, musicians and local schools. The song predominately focuses on the key steps involved in effective handwashing. The key steps of running hands under clean water, rubbing hands together, using soap, scrubbing in between fingers, under nails, around wrists and drying hands are accompanied by actions which the children are encouraged to follow along with. The song utilises several of the behaviour change techniques outlined by Michie and colleagues [51], such as providing instructions on how to perform handwashing correctly and behavioural rehearsal through the repetitive practising of the handwashing actions. Furthermore, co-creating the song alongside school children ensured that the terminology and delivery of the song is accessible to the intended age group. Musical interventions have been utilised successfully in prior health education interventions such as malaria [52] and are particularly suitable for the young EYFS age range to facilitate memory and learning [53]. The associated video also reinforces the song by depicting the lyrical content (e.g., depicting children washing their hands). The group of children depicting the song lyrics in the video are of a similar age range to the intended age of the intervention recipients. The literature proposes that observers are more likely to imitate models that are similar to them, with prior studies indicating that similarity in age and group modelling of behaviour rather than a singular model is indicative of behaviour adoption [54, 55].

#### Website: Interactive online games

The third resource is a set of interactive web-based games aiming to deepen children’s understanding of contact and contamination, again targeting the psychological capability element of the COM-B model via the education function. Due to the ubiquity of technological devices, educational games are progressively being used in health-related areas to instigate behavioural change [56], and a prior content analysis has called for the use of more technology in HH interventions [24]. The use of technological games in a HH intervention can capitalise on children’s pre-existing preference and intrinsic motivation towards playing online games [57]. Additionally, the interactive nature and continuous feedback that games provide can result in increased engagement, which is important for the retention of information [58].

#### Glo-gel: Experiential small group activity

The fourth resource is an interactive activity in which children are asked in small groups to rub a small amount of glo-gel onto their hands. They are then asked to wash their hands and place their hands under a UV light, which shows (i.e., “glows”) areas that the child may have missed and they can compare their hands with their peers. This again reinforces the concept of invisible germs and highlights to the children key areas they may be missing in their current handwashing practices. A recent study has shown that the use of glow-gel as an educational tool led to improvements in handwashing quality in pre-school age children [59]. As the activity is completed in small groups, it also has the potential to target the social opportunity element of the COM-B model, as individuals may feel social pressure to wash their hands effectively, ensuring to cover all areas.

### Aims of the educational intervention

In the current research, two intervention studies aimed to evaluate the efficacy of A Germ’s Journey educational resources. In Study 1, the resources were combined into a multicomponent intervention that was delivered in schools. Multicomponent interventions targeting multiple elements of the COM-B model are predicted to be most effective at improving behaviour change outcomes [41]; thus, it was hypothesised that this intervention would yield significant improvements in both handwashing quality and understanding of germs. In Study 2, the song activity was delivered as an intervention in a public space, the Thinktank Birmingham Science Museum, emphasising a particular component of the resources and assessing its utility in a less controlled public environment. Song-based interventions have previously been shown to improve behaviour change outcomes [52]; thus, it was hypothesised that this intervention would also yield significant improvements in handwashing quality.

## Study 1: Intervention in school-based settings

In Study 1, the book, song, web-based games and glo-gel activities were combined into a multicomponent intervention that was delivered in schools as a structured carousel workshop, utilising the service provision element of the BCW. Each activity was delivered by a researcher and the children moved among the activities in small groups. The concept of the workshop design is underpinned by the theory of experiential learning, which refers to constructing knowledge from experiences [60]. Prior research has indicated that HH is particularly susceptible to normative influence due to it being a publicly enacted behaviour [61]. The group environment of the intervention facilitated the social opportunity element of the COM-B model. By providing the intervention to a whole class, the intervention had the potential to target both the children’s descriptive and injunctive norms. For example, the intervention can result in HH improvements among members of the class and when individual children observe improvements in their peers’ HH, this may encourage them to wash their hands effectively and further improve the effectiveness of the intervention (descriptive norms). Similarly, all of the children in the class will have been taught about the importance of effective HH at the same time. This may create a belief among the children that other members of their class expect them to carry out effective HH behaviours, encouraging them to conform to the norm (injunctive norms).

## Materials and methods

### Participants

225 children from four primary schools in Leicestershire, England were recruited to participate. The children were all enrolled in EYFS classes. The schools were located in Leicester city centre (2; n = 117) and rural Leicestershire (2; n = 108). The sample enabled detection of an effect size of *d*_*z*_ = 0.19 via a paired comparison (e.g., baseline vs. follow-up; power = .80, α = .05). The study was not preregistered. Informed written consent was provided by the head teacher at each school, acting in loco-parentis. In addition, parents and guardians were provided an informational letter that explained the research. Consent was provided by parents and carers via an opt-out approach, which provided them with the option to withdraw their child from the observations. The research was approved by De Montfort University’s Health and Life sciences Research Ethics Committee and adhered to the British Education Research Association ethical code of practise. Finally, confidentiality was maintained.

#### Design

The study used a cluster randomised control design. At each school, one class was randomly assigned to the intervention group and one class was randomly assigned to the control group (i.e., between participants). One school included three rather than two classes; children from one of these classes were randomly split between the two groups. Two sessions were scheduled at each school, spaced approximately one month apart. In the intervention group (n = 120), observations were made at baseline and post intervention in the first session and at follow up in the second session. In the control group (n = 105), observations were made at baseline in the first session and at follow up in the second session. The study was analysed using regression and related analyses.

#### Materials

During each observation (e.g., baseline, post intervention and follow up), both handwashing quality and knowledge were assessed. Handwashing quality was assessed using a six-item observation checklist. Two researchers per child recorded whether or not participants used soap (soap), rubbed their hands together (rub), made contact with their wrists (wrists), made contact in between their fingers (fingers), made contact under their nails (nails) and/or dried their hands (dry). Knowledge was assessed by asking participants, “Why do we wash our hands?” Researchers recorded whether or not participants’ responses included “germs”. Finally, the intervention made use of the book, song, games and glo-gel activities.

#### Procedure

Teachers assigned participants unique numbers, which they wore as stickers (e.g., enabling their data to be linked across observations). During each observation, participants were asked to wash their hands while they were observed by two researchers each, who recorded their observations using the six-item behaviour checklist (i.e., handwashing quality). Participants were also asked, “Why do we wash our hands?” (i.e., knowledge).

During the first session, baseline observations were made of both the intervention and control groups. In addition, the researchers delivered the structured carousel workshop to the intervention group. First, participants took part in the song activity as a whole class. Second, the participants took part in each of the activities, reading the book, playing the games and partaking in the Glo-gel rubbing and washing off and moving around each of these tasks in small groups. Third, participants again took part in the song activity as a whole class. The workshop lasted approximately 40 minutes in total. Finally, post intervention observations were made of the intervention group. Conversely, participants in the control group took part in activities unrelated to HH. These sessions were all scheduled in the morning.

During the second session, follow up observations were made of both the intervention and control groups. For each pair of observers per participant in these sessions, one was not present at the first session, and was thus blind to group. Finally, teachers were provided with the intervention materials (e.g., to engage the control group). This also addresses the ethical disparity and moral dimension regarding the control group missing out on the intervention and the learning opportunities afforded by the handwashing workshop delivered to the intervention group. These sessions were all scheduled approximately one month after the first session.

### Results

Both participants’ behavioural and knowledge outcomes were analysed. Participants with missing data (n = 32), particularly those who were absent from one of the sessions, were excluded. Analyses were run using R version 3.6.1; α = 0.05 and 95% confidence intervals are reported. Cohen’s kappa indicated strong agreement between pairs of observers (kappa = 0.84; *CI*: 0.82, 0.86). For the analyses, participants were coded as engaging in a binomial handwashing behaviour when both observers were in agreement. In addition, a total behavioural score was generated by summing the number of handwashing behaviours observed for each participant. Finally, a binomial knowledge score was generated based on whether participants’ responses included “germs” as a reason for washing their hands.

#### Baseline vs. post intervention

**Fig 1:**
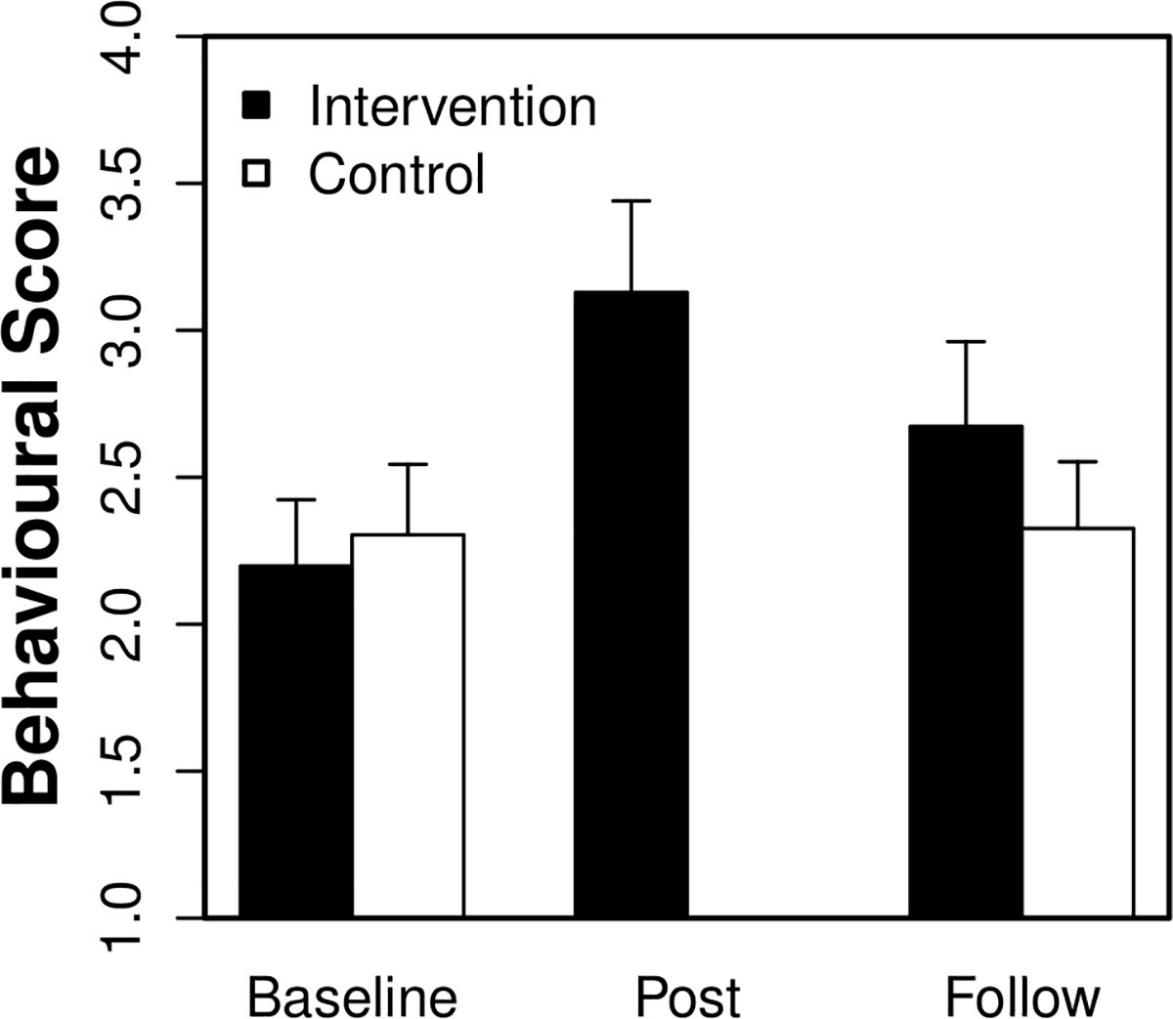
Study 1: Mean (95% *CI*) behavioural total scores in the intervention and control groups. The maximum score of 6 reflects engagement in all of the handwashing behaviours observed (i.e., soap, rub, wrists, fingers, nails and dry).

First, the intervention group (n = 101) was compared between baseline vs. post intervention. Table 2 reports the percentages of participants at baseline vs. post intervention who demonstrated each handwashing behaviour and whose responses included “germs”. Fig 1 reports participants’ mean behavioural total scores. The behavioural and knowledge scores were submitted to mixed effects models (lme4 [55]) with random intercepts for both schools and participants and fixed effects of observation (baseline = -0.50; post = 0.50). Throughout, random slopes were excluded from models due to issues with convergence. Significant improvements between baseline vs. post intervention were observed for both behavioural scores, *Est* = 0.93, *SE* = 0.14, *t* = 6.57, *p* < 0.001, and knowledge scores, *Est* = 3.12, *SE* = 0.81, *z* = 3.87, *p* < 0.001. Due to low event occurrences (e.g., see wrists and nails behaviours), handwashing behaviours were not submitted individually to mixed effects analyses. McNemar's chi-squared tests with continuity correction (mid-*p* tests are also reported; see Table 2) revealed significant improvements between baseline vs. post intervention for soap, wrists, fingers and nails behaviours. Estimated odds ratios, reflecting the number of positive (i.e., not observed to observed) over negative (i.e., observed to not observed) changes, are also reported.

**Table 2:** Study 1 baseline vs. post intervention: Percentages of participants in the intervention group (n = 101) who demonstrated each handwashing behaviour and whose responses included “germs”.

Measure	Baseline	Post	McNemar Test			
			χ^2^	*p*	mid-*p*	Estimated
	% (n)	% (n)				Odds Ratio (*CI*)
Soap	55% (56)	71% (72)	10.23	0.001	< 0.001	6.33 (1.86, 33.42)
Rub	70% (71)	76% (77)	0.89	0.34	0.26	1.55 (0.68, 3.65)
Wrists	4% (4)	29% (29)	21.33	< 0.001	< 0.001	26.00 (4.27, 1065.94)
Fingers	11% (11)	34% (34)	16.69	< 0.001	< 0.001	8.67 (2.66, 44.74)
Nails	1% (1)	19% (19)	14.45	< 0.001	< 0.001	19.00 (3.02, 789.46)
Dry	78% (79)	84% (85)	1.14	0.29	0.21	1.75 (0.69, 4.81)
Knowledge	35% (35)	75% (76)	37.21	< 0.001	< 0.001	42.00 (7.14, 1697.91)

The analyses compare baseline and post intervention measures within the intervention group.

#### Intervention vs. control

Second, the intervention (n = 101) and control (n = 92) groups were compared between baseline vs. follow up. Table 3 reports the percentages of participants at baseline vs. follow up in both the intervention and control groups who demonstrated each handwashing behaviour and whose responses included “germs”. Fig 1 again reports participants’ mean behavioural total scores. The behavioural and knowledge scores were submitted to mixed effects models with random intercepts for schools, classrooms and participants and fixed effects of both observation (baseline = -0.50; follow = 0.50) and group (intervention = -0.50; control = 0.50). Significant interactions between observation and group were observed for both behavioural scores, *Est* = -0.45, *SE* = 0.19, *t* = -2.44, *p* = 0.01, and knowledge scores, *Est* = -1.81, *SE* = 0.59, *z* = -3.08, *p* = 0.002.

**Table 3:** Study 1 baseline vs. follow up: Percentages of participants in the intervention and control groups who demonstrated each handwashing behaviour and whose responses included “germs”.

Measure	Baseline	Follow	McNemar Test			
			χ^2^	*p*	mid-*p*	Estimated Odds
	% (n)	% (n)				Ratio (*CI*)
	**Intervention Group** (n = 101)					
Soap	55% (56)	61% (62)	1.25	0.26	0.19	1.86 (0.69, 5.50)
Rub	70% (71)	67% (68)	0.12	0.73	0.61	0.83 (0.39, 1.75)
Wrists	4% (4)	16% (16)	8.64	0.003	0.001	13.00 (1.95, 552.47)
Fingers	11% (11)	29% (29)	9.63	0.002	< 0.001	4.00 (1.59, 11.96)
Nails	1% (1)	10% (10)	5.82	0.02	0.006	10.00 (1.42, 433.98)
Dry	78% (79)	84% (85)	0.96	0.33	0.25	1.60 (0.68, 3.94)
Knowledge	35% (35)	65% (66)	24.32	< 0.001	< 0.001	11.33 (3.56, 57.67)
	**Control Group** (n = 92)					
Soap	61% (56)	57% (52)	0.50	0.48	0.36	0.64 (0.21, 1.80)
Rub	65% (60)	70% (64)	0.45	0.50	0.38	1.50 (0.56, 4.23)
Wrists	4% (4)	4% (4)	0.00	1.00	1.00	1.00 (0.19, 5.37)
Fingers	16% (15)	16% (15)	0.00	1.00	1.00	1.00 (0.35, 2.84)
Nails	1% (1)	0% (0)	0.00	1.00	0.50	0.00 (0.00, 39.00)
Dry	83% (76)	86% (79)	0.24	0.63	0.48	1.43 (0.49, 4.42)
Knowledge	47% (43)	52% (48)	0.64	0.42	0.33	1.50 (0.63, 3.73)

The analyses compare baseline and follow up measures within each group.

In order to unpack these interactions, the intervention and control groups were compared separately between baseline vs. follow up using planned pairwise comparisons. The behavioural and knowledge scores were submitted to mixed effects models with random intercepts for both schools and participants and fixed effects of observation (baseline = -0.50; follow = 0.50). In the intervention group, significant improvements between baseline vs. follow up were observed for both behavioural scores, *Est* = 0.48, *SE* = 0.14, *t* = 3.30, *p* = 0.001, and knowledge scores, *Est* = 2.14, *SE* = 0.52, *z* = 4.11, *p* < 0.001. In contrast, no differences were observed in the control group (*t*s < 1). In addition, McNemar's tests revealed significant improvements between baseline vs. follow up for wrists, fingers and nails behaviours in the intervention group. In contrast, no differences were observed in the control group.

#### Knowledge predictor

Finally, participants in the intervention group whose responses included “germs” post intervention were compared to participants in the intervention group whose responses did not. Independent samples *t*-tests revealed significant differences in the behavioural total scores of these two groups both post intervention (germs: *M* = 3.30, *SD* = 1.64; not: *M* = 2.60, *SD* = 1.35), *t*(49.13) = 2.13, *p* = 0.04 (*CI*: 0.04, 1.37), and at follow up (germs: *M* = 2.89, *SD* = 1.47; not: *M* = 2.00, *SD* = 1.29), *t*(46.30) = 2.90, *p* = 0.006 (*CI*: 0.27, 1.52). Knowledge of “germs” was associated with engagement in more handwashing behaviours, which persisted from post intervention through follow up.

## Study 2: Intervention in public spaces

In Study 2, the song activity was delivered as an intervention in a public space, the Thinktank Birmingham Science Museum. The song activity was created collaboratively with the museum and is also incorporated into the museum’s MiniBrum exhibit, which was designed for children under eight years of age. The museum has welcomed over 100,000 visitors since the opening of the MiniBrum exhibit. A video monitor in the exhibit’s toilets, which is integrated into the mirror in front of the sinks, plays the song and its associated video. In contrast to Study 1, Study 2 aimed to evaluate the efficacy of this song activity in isolation, providing an understanding of how a specific component of the resources contributes to its success outside a school setting.

## Materials and methods

### Participants

104 children who visited the museum during a one-week period were recruited to participate. Children reflected a wider range of ages (*M* = 6.54, *SD* = 2.27; Range = 3 to 12) than the EYFS pupils included in Study 1. The sample enabled detection of an effect size of *d* = 0.55 via an independent comparison (e.g., intervention vs. control; power = .80, α = .05). The study was not preregistered. Ethical clearance was provided by the museum and informed written consent was provided by parents and guardians of the participating children. The research was approved by De Montfort University’s Health and Life Sciences Research Ethics Committee and adhered to the British Education Research Association ethical code of practise. Finally, confidentiality was maintained.

#### Design

The study used a randomised control design. Participants were randomly assigned to the intervention or control group (i.e., between participants). A single observation was made of children in both groups, either after (i.e., intervention; n = 54) or before (i.e., control; n = 50) taking part in the song activity.

#### Materials

Again, handwashing quality was assessed using the six-item observation checklist, and knowledge was assessed by asking participants, “Why do we wash our hands?” Finally, the intervention made use of the song activity.

#### Procedure

The research team was located at an informational stand alongside the museum’s MiniBrum exhibit, where they invited parents and guardians and their children to participate in the research. Participants took part in the song activity that is integrated into the exhibit. As in Study 1, participants were also asked to wash their hands while they were observed by two researchers, who recorded their observations using the six-item behaviour checklist (i.e., handwashing quality). Participants were also asked, “Why do we wash our hands?” (i.e., knowledge). While participants in the intervention group took part in the song activity first followed by the observation second, participants in the control group took part in the observation first followed by the song activity second. Thus, all participants took part in the song activity and were observed once, but the control group provided a pre intervention baseline. The sessions lasted approximately 5 minutes in total.

### Results

Again, both participants’ behavioural and knowledge outcomes were analysed. Participants with missing data (n = 4) were excluded. Participants were observed by two researchers (Cohen’s kappa = 0.83; *CI*: 0.78, 0.87) and coded as engaging in a behaviour when both observers were in agreement.

#### Age predictor

First, age-related effects were analysed. In contrast to Study 1, Study 2 included a wider range of ages, including a number of participants who were eight years of age and older (n = 28). Pearson’s and point-biserial correlations, respectively, revealed significant relationships between age and both behavioural total scores, *r*(98) = 0.37, *p* <0.001 (*CI*: 0.18, 0.53), and knowledge scores, *r*(98) = 0.21, *p* = 0.03 (CI: 0.02, 0.39). Generally, older children engaged in significantly more handwashing behaviours and showed significantly greater knowledge of “germs”. In order to more closely capture the age group that the exhibit was designed for, and to more closely align with Study 1, only participants under eight years of age were included in the analysis (n = 72).

#### Intervention vs. control

**Fig 2:**
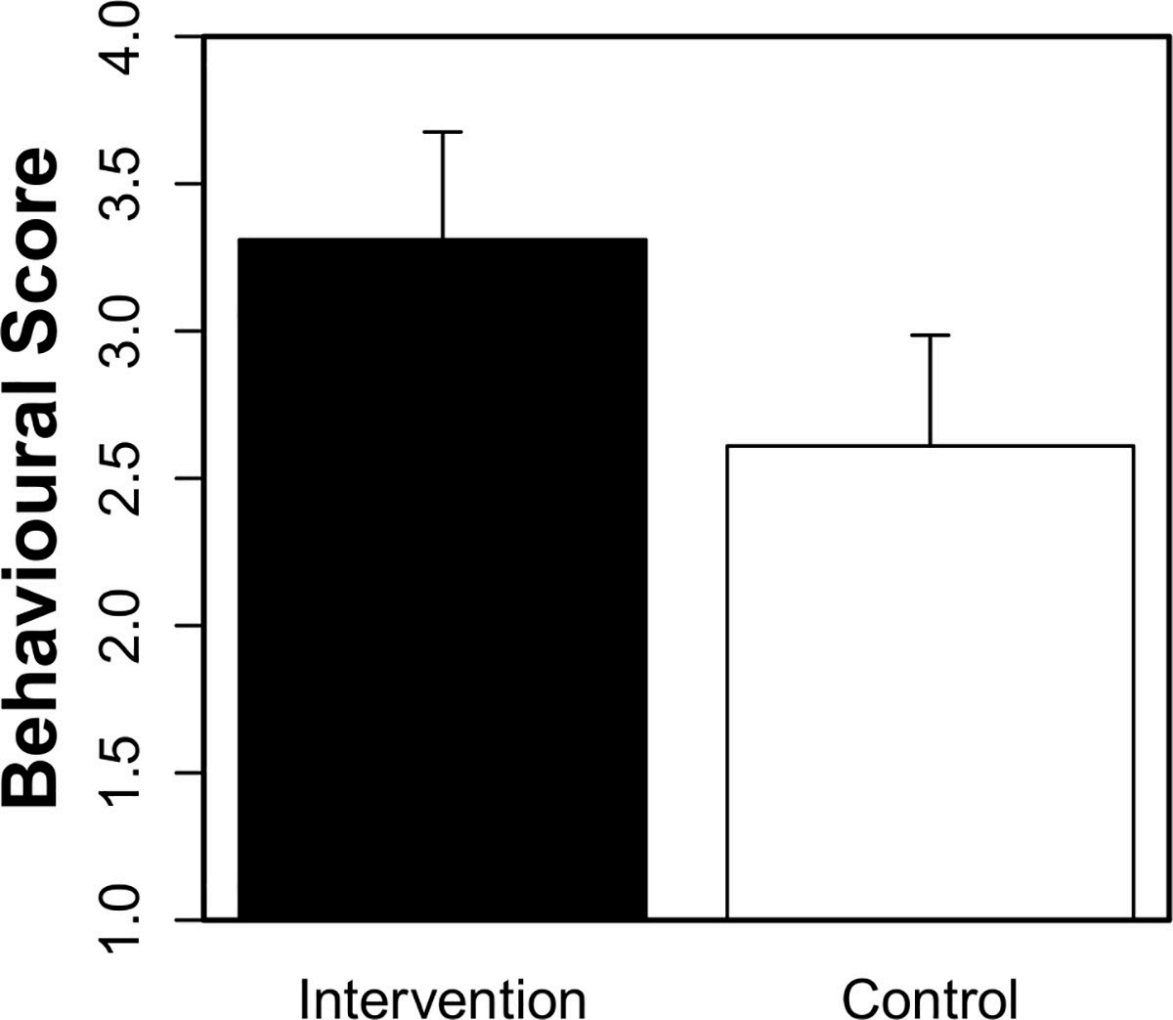
Study 2: Mean (95% *CI*) behavioural total scores in the intervention and control groups. The maximum score of 6 reflects engagement in all of the handwashing behaviours observed (i.e., soap, rub, wrists, fingers, nails and dry).

Second, the intervention (n = 36) and control (n = 36) groups were compared whilst excluding participants eight years of age and older. An independent samples *t*-test revealed that age did not differ significantly between the intervention (*M* = 5.47, *SD* = 1.23) and control (*M* = 5.47, *SD* = 1.18) groups, *t*(69.89) = 0.00, *p* = 1.00 (*CI*: -0.57, 0.57). Table 4 reports the percentages of participants in both the intervention and control groups who demonstrated each handwashing behaviour and whose responses included “germs”. Fig 2 reports participants’ mean behavioural total scores. The behavioural and knowledge scores were submitted to regression models with predictors of group (intervention = -0.50; control = 0.50) and age (centred) and their interaction. For the behavioural scores, group (*Est*. = -0.71, *SE* = 0.34, *t* = -2.07, *p* = 0.04) and age (*Est*. = 0.87, *SE* = 0.23, *t* = 3.71, *p* < 0.001) (but not their interaction, *t* < 1) were significant, such that scores were both higher in the intervention than control group and increased with age. For the knowledge scores, group (*Est*. = -2.56, *SE* = 1.18, *z* = -2.18, *p* = 0.03), age (*Est*. = 1.99, *SE* = 0.65, *z* = 3.04, *p* = 0.002) and their interaction (*Est*. = -2.57, SE = 1.31, z = -1.97, p = 0.05) were significant, such that particularly among older participants, scores were higher in the intervention than control group. Chi-squared tests with continuity correction (see Table 4) also revealed significantly higher engagement in fingers behaviours in the intervention than control group.

**Table 4:** Study 2 intervention vs. control: Percentages of participants in the intervention (n = 36) and control (n = 36) groups under eight years of age who demonstrated each handwashing behaviour and whose responses included “germs”.

Measure	Intervention %	Control %	Chi-squared Test			
			χ^2^	*p*	Fisher's *p*	Estimated Odds
	(n)	(n)				Ratio (*CI*)
Soap	83% (30)	81% (29)	0.09	0.76	1.00	1.20 (0.30, 4.91)
Rub	94% (34)	86% (31)	1.42	0.23	0.43	2.71 (0.41, 30.35)
Wrists	8% (3)	0% (0)	3.13	0.08	0.24	7.63 (0.08, 752.80)*
Fingers	53% (19)	25% (9)	5.84	0.02	0.03	3.29 (1.11, 10.38)
Nails	8% (3)	0% (0)	3.13	0.08	0.24	7.63 (0.08, 752.80)*
Dry	83% (30)	69% (25)	1.93	0.17	0.27	2.18 (0.63, 8.25)
Knowledge	75% (27)	61% (22)	1.60	0.21	0.31	1.89 (0.62, 5.99)

*Haldane-Anscombe correction.

The analyses compare the intervention and control groups.

## Discussion

The current study evaluated the impact of A Germ’s Journey educational resources on young children’s knowledge and behaviour. These resources were designed to support young children in learning about germs and handwashing, and include a book, website, song, online games and glo-gel activities. Building on prior research, both knowledge and behavioural outcomes were measured and compared to a control group.

Study 1 aimed to evaluate the impact of a multicomponent behaviour change intervention on young children’s HH in a school setting. In order to maximise its potential for success, the intervention was grounded in psychological behaviour change theory (BCW [36]), and integrated the book, song, web-based games and glo-gel activities from A Germ’s Journey educational resources. The intervention focused on the quality of the handwashing and included both practical (how to wash hands) and conceptual (understanding of germ transfer) aspects. In comparison to the control group, the results showed that children who received the intervention improved both their handwashing practices and their knowledge of germs. The average number of handwashing behaviours observed (i.e., total behavioural scores; see Fig 1) increased from 2.20 at baseline to 3.13 at post intervention and 2.67 at follow up, and the number of children mentioning germs in their responses increased between baseline and post intervention by 40% and between baseline and follow up by 30%.This demonstrates that the improvement was apparent immediately after the intervention, and crucially, remained significant following a one month delay. In particular, children improved significantly on washing in between their fingers (up 23% at post intervention and 18% at follow up relative to baseline), around their wrists (up 25% at post intervention and 12% at follow up) and under their nails (up 18% at post intervention and 9% at follow up) (see Tables 2 and 3). Although Dingman and colleagues [59] also found significant improvement in overall handwashing behaviour following an intervention, here we showed that our intervention led to improvements in washing different areas of the hands over a sustained period of time. This is important as targeting and contacting key areas of the hand is essential to ensure effective handwashing and the coverage of all hand skin surfaces [62] The activities in our intervention were designed to reinforce each other, were multifaceted, and combined different elements of the COM-B model [36]. Taken together, these factors supported an effective intervention that yielded a range of HH improvements [35].

However, the study also revealed the need for further improvement; for example, only 10% of children washed under their nails at follow up, and no behaviour reached 100%. However, given the very young age range of the children (e.g., 4–5 years old), this was not unexpected or surprising Therefore, in order to increase the impact of the intervention further, we would recommend repeating each activity at regular intervals. This would increase the opportunity for learning and reinforce children’s motivation. For example, the handwashing song, which explains key areas to wash, could be played before lunch *daily* before children are asked to wash their hands. In addition, teachers and parents could be encouraged to observe children’s handwashing to provide praise and feedback and hence further increase children’s motivation to improve the quality of their handwashing [51, 22]. Finally, in many instances children applied soap but rinsed it off before rubbing their hands together; repetition would also likely support the appropriate ordering of actions.

Study 2 aimed to evaluate the impact of the song activity as a single isolated component on young children’s HH. This study served the purpose of examining the immediate impact of the handwashing song in a public space. In contrast to the control group, the results showed that overall, young children’s handwashing behaviour improved after taking part in the song activity. The average number of handwashing behaviours observed (i.e., total behavioural scores; see Fig 2) was 3.31 in the intervention group vs. 2.61 in the control group. In particular, washing in between the fingers improved significantly (53% of participants did so in intervention group compared to 25% of participants in the control group). This demonstrates that despite being delivered in a less controlled public environment, the intervention can still lead to positive improvements in young children’s handwashing practices.

Nevertheless, while the song activity on its own (Study 2) had a positive impact, the structured carousel workshop (Study 1) impacted more aspects of handwashing. The enhanced effects of the carousel are consistent with the COM-B model, which predicts that interventions targeting multiple elements are likely to be more effective. The song predominately targeted the capability element of the COM-B model by educating and training children on key handwashing steps. Other components of the resources bring in further elements of the COM-B model (e.g., motivation in the book and social opportunity from the delivery to the whole class), which may account for this difference.

Furthermore, the carousel also yielded improvements in young children’s understanding of germs and germ transfer, which may have further reinforced the behavioural changes. This is supported by Study 1, which found that participants’ knowledge of germs was associated with engagement in more handwashing behaviours both at the post intervention stage and follow up. Having an understanding of germ transfer is likely to target the reflective motivation element of the COM-B model by increasing the children’s perception of risk to germs and encouraging them to engage in the protective behaviour of effective handwashing. This demonstrates the importance of targeting children’s knowledge of germ transfer in order to increase the likelihood of HH behavioural change.

Taken together, the results from these two studies support the application of the BCW in intervention design. They demonstrate that the model can be applied successfully in the context of hand-hygiene behaviour change. Additionally, the studies provide evidence for the claim that interventions targeting multiple elements of the COM-B model are most effective [41] as the multi-component carousel of activities used within Study 1 were more effective than the song based activity alone (Study 2). The utilisation of the BCW strengthened the intervention by providing it with a theoretical backing and facilitated in a comprehensive analysis of the behavioural determinants of effective handwashing in young children. This meant that multiple components of the COM-B model could be targeted to ensure the interventions success, which is especially important given the complex and multifactorial nature of hand hygiene behaviours. Finally, the current results suggest that the model provides a powerful tool for understanding the determinants of behaviour across a diversity of novel behaviours and contexts.

In future research it will be important to evaluate children’s conceptual understanding of germs in more detail. Here, we asked a single question (i.e., “Why do we wash our hands?”) and recorded whether participants’ responses included germs or not. A different approach might be to ask children what germs are, and to use a comparative judgement approach to evaluate their conceptual understanding [e.g., 63]. In a previous study [47] conducted in India, identical pre and post-intervention worksheets were developed in order to evaluate the direct impact that A Germ’s Journey resources had on children’s knowledge of germ transmission and handwashing. Although this method allowed for a more comprehensive insight into the children’s understanding, due to time constraints it was beyond the scope of this study. Relatedly, it will also be important to evaluate the impacts of other participant characteristics. Although demographic information about participants’ (e.g., ethnic, language, etc.) backgrounds was not collected in Study 1 or 2, the two city schools in Study 1 were located in diverse areas (e.g., based on ward data from the 2011 Census, these areas were approximately 20% and 80% white, with approximately 25% and 10% of households reporting that English was not their main language) that contrasted with the rural schools (e.g., these areas were over 90% white, with over 95% of households reporting that English was their main language). The schools were not compared directly (e.g., again, demographics were not collected from participants directly), but both city (baseline: *M* = 1.87, *SD* = 1.09; post: *M* = 2.72, *SD* = 1.59) and rural (baseline *M* = 2.47, *SD* = 1.15; post *M* = 3.47, *SD* = 1.54) schools showed numerical improvements in the intervention group, suggesting that the intervention is suitable across a diversity of backgrounds.

Children’s use of soap and the rubbing and drying of their hands did not change in either study. One reason for this may be because these behaviours showed higher baseline rates (e.g., soap: 55% in Study 1 and 83% in Study 2; rub: 70% in Study 1 and 94% in Study 2; dry: 78% in Study 1 and 83% in Study 2). Therefore, participants were already practising these behaviours at a higher rate compared to the other behaviours before the intervention, which left a smaller amount of room for improvement. Potentially, changes in the environment (physical opportunity) might impact these behaviours further [22]. For example, in many schools as well as at the museum, the soap dispensers were difficult to reach for some children (e.g., too high on the wall) or difficult to see (e.g., positioned behind the door). It may be that children need to be cued as to where the soap dispensers and hand drying facilities are and how to reach them [35]. In addition, some children avoided drying their hands because of the noisy hand driers, hence the use of paper towels may be more user friendly for children, as well as being more hygienic (for review, see [64]). These environmental factors fall under the physical opportunity element of the COM-B model, which was not targeted in the current intervention due to restrictions on the study locations. Prior research has suggested that simple environmental changes can lead to improved outcomes in HH behaviours. For example, the presence of paper towels and visual cues such as arrows and picture of eyes above sinks can lead to improvements in HH outcomes [65–67]. Reflecting this, the current results would likely be bolstered by supplementing the current intervention resources with further environmental changes.

## Conclusions

In conclusion, prior research has shown that improved HH leads to a reduction in infectious disease (for review, see [68]). Despite challenges young children present for improving HH, this study found that A Germ’s Journey educational resources, which targeted the quality of handwashing in young children and their understanding of germs, were successful in improving handwashing practices. This study made a significant impact on both young children’s knowledge of germ transfer and handwashing quality, that was sustained over a one month time period. For example, approximately one month after the intervention in Study 1, 30% more children linked germs to handwashing and the average number of handwashing behaviours observed was significantly higher at the follow up visit (2.67) compared to baseline (2.20). Likewise, in Study 2, which focussed solely on the song activity, those that watched the song/video were observed engaging in on average 3.31 of the 6 handwashing behaviours compared to 2.61 in the control group. A Germ’s Journey educational resources provided an effective resource for improving HH and preventing the spread of infectious disease in children, two important public health concerns, particularly in light of the current Covid-19 pandemic. As outlined by the WHO, handwashing remains the key strategy for reducing and containing the transmission of COVID-19 and other infectious diseases [3, 68].
